# Developing and Evaluating a WeChat-Based Applet Fluid Intake Reminder on Enhancing Fluid Adherence in Postoperative Patients With Urinary Calculi: Protocol for a Randomized Controlled Trial

**DOI:** 10.2196/80214

**Published:** 2026-02-19

**Authors:** Xue Zhou, Tongyao Wang, Qian Chen

**Affiliations:** 1 School of Nursing, Li Ka Shing Faculty of Medicine, University of Hong Kong, Room 532, Academic Building at No 3, Sassoon Road, Pokfulam, Hong Kong, China (Hong Kong), 852 39102790; 2 Department of Urology, Shenzhen Qianhai Shekou Free Trade Zone Hospital, Shenzhen, China

**Keywords:** fluid adherence, urinary calculi, digital therapeutic, WeChat applet, fluid reminder

## Abstract

**Background:**

Urinary calculi (UC), affecting 1%‐13% globally, pose a significant health burden due to high recurrence rates (up to 50% within 10 years) and substantial health care costs. Adequate fluid intake is a cornerstone of prevention; yet, its adherence remains poor due to forgetfulness, lifestyle barriers, and limited patient education. Existing mobile health interventions for UC prevention often lack medical oversight and clinical validation. WeChat-based digital therapeutic intervention may have a positive effect on fluid adherence in this patient group.

**Objective:**

Our objective is to develop a WeChat applet to improve hydration behavior and reduce stone recurrence among postoperative patients with UC.

**Methods:**

This is an open-label, 2-arm, parallel-group randomized controlled trial. We will recruit 148 participants from China’s tertiary hospital and randomly allocate them in a ratio of 1:1 to the intervention or control group. The intervention group received standard postoperative care supplemented by the WeChat-Based Applet Fluid Intake Reminder (WAFIR), which delivers personalized fluid intake reminders, urine color monitoring, 24-hour fluid intake and urine output tracking, and evidence-based educational content on hydration and urolithiasis management. The control group receives standard care of general discharge instructions from nurses. The primary outcome is the fluid adherence, measured by 24-hour fluid intake and urine volume; secondary outcome measures are the Wisconsin Stone Quality of Life Questionnaire, Patient Health Questionnaire-9, Electronic Health Literacy Scale, physical activity (International Physical Activity Questionnaire—Short Form), and recurrence rate of UC. Outcomes are measured before intervention (T0) and after a 1-month (T1) and 3-month (T2) follow-up period. Intention-to-treat analysis, 2-tailed *t* tests, and repeated measures ANOVA will be used to compare outcomes; statistical significance is set at a *P*<.05 significance threshold. The study was approved by the ethics review board in December 2024.

**Results:**

The development of WAFIR, conducted in collaboration with stakeholders, was finalized in February 2025. Recruitment commenced on March 1, 2025; data collection was completed in September 2025, and data analysis was analyzed in December 2025. Dissemination of findings is planned through conferences and publications in 2026.

**Conclusions:**

This research evaluates the effectiveness of a nurse-led, evidence-based digital therapeutic intervention, WAFIR, in overcoming fluid adherence barriers among postoperative patients following urolithiasis surgery, aiming to increase daily fluid intake and urine output, reduce recurrence rates, enhance quality of life, and generate empirical evidence for its application in urology care, thereby optimizing postoperative management within clinical settings.

## Introduction

Urolithiasis, or urinary calculi (UC), is emerging as a significant global health crisis, affecting individuals of all ages and genders. The prevalence of UC has been increasing worldwide over the past decades [1]. Estimated incidence rates are 7%‐13% in North America, 5%‐9% in Europe, and 1%‐5% in Asia [2]. In low-income countries, up to 25% of the population can be affected, often with fatal outcomes due to inadequate urological care [3]. In China, the prevalence of nephrolithiasis is around 5.8%, exceeding 10% in the southern region [4]. Meanwhile, the high recurrence rate of UC poses a significant challenge, with approximately 10%, 30%, and 50% of individuals experiencing recurrence at 1 year, 5 years, and 10 years, respectively [5-7]. The financial burden of UC is substantial, with yearly medical expenses estimated at 10 billion dollars in the United States and US $695.51 million in Europe [8,9]. Predictive models indicate that the high prevalence and recurrence rate will lead to a 25% increase in health care costs over the next 50 years [10]. This makes UC one of the most expensive urological diseases, highlighting the urgent need for better understanding and management of this condition.

The occurrence of UC is influenced by a variety of environmental, climatic, and dietary factors. Environmental and climatic conditions, particularly in hot climates, can contribute significantly to water loss and water quality issues. High temperatures often lead to increased perspiration, which in turn reduces overall water levels in the body. This dehydration can concentrate the urine, increasing the likelihood of stone formation. Additionally, water quality, specifically the levels of sodium and other minerals, plays a crucial role in this process [11,12]. Dietary habits are another critical factor in the development of urinary stones. Insufficient water intake is one of the primary dietary risk factors, as it leads to more concentrated urine, which facilitates the crystallization process. Poor dietary habits, such as the prolonged consumption of oxalate-rich foods—like spinach, nuts, and chocolates—significantly elevate the propensity for developing calcium oxalate stones [13]. High-sugar and high-fat diets further exacerbate this risk by influencing urinary elimination processes, making the urine more conducive to stone formation [14]. These risk factors highlight the important role of nurses in implementing targeted hydration protocols and dietary interventions to mitigate stone recurrence.

The European Association of Urology and the American Urological Association both strongly emphasize the importance of increasing fluid intake to prevent urinary stone formation. Research indicates that increasing fluid consumption stands out as a simple and economically feasible way to lower the risk of stone formation [15]. Then, McCauley et al [16] discovered that patients tend to be more open to increasing fluid intake than adopting other dietary or pharmaceutical therapies. Yet, Khambti et al [17] identified a challenge, noting that only about half of patients with urinary stones adhere to hydration recommendations, as determined through 24-hour urine output tests. Building on this, further research elucidated the underlying causes: at first, patients might not know or remember to drink enough; then, some dislike the taste of water or do not feel thirsty; and finally, the need for frequent voiding or workplace factors gets in the way [16]. Other research backs this up, showing that forgetfulness or not realizing their need for hydration plays a big role in poor fluid habits [18,19]. Therefore, enhancing adherence to fluid intake recommendations offers substantial benefits, including reduced health care expenditure through lower incidence of urinary stones and associated costs, improved public health outcomes, and an elevated quality of life (QoL) by decreasing the frequency and severity of symptoms.

Digital therapeutic interventions (DTx) deliver evidence-based, software-driven solutions via mobile apps, wearables, and web platforms to manage conditions. Unlike wellness tools, DTx is clinically validated, often regulated, and designed for measurable health outcomes [20]. By 2026, the global mobile app market was expansive, with approximately 4 million apps available on the Apple App Store and Google Play, reflecting the vast potential of digital health tools [21]. However, conventional mobile apps face significant barriers to widespread adoption, such as complex development processes, protracted timelines, and substantial financial investment, which often limit their scalability and accessibility in clinical settings. In contrast, WeChat applets, which require no installation and are integrated into China’s predominant social media platform, facilitate seamless, on-demand access, effectively addressing technical barriers and offering distinct advantages by enhancing user convenience and minimizing operational challenges. With over 1.4 billion monthly active users, WeChat has a vast reach and engagement, making it a widely used communication tool in China. Using WeChat’s existing infrastructure, the applets simplify the process by reducing the need for extensive coding and platform-specific adaptation and allow individuals to access them instantly. The significance of WeChat in health care has been amplified by its rapid adoption during the COVID-19 pandemic, which catalyzed its integration into various medical contexts, from telemedicine to patient education, markedly improving care quality [22,23]. Notably, the feasibility of using WeChat to enhance adherence to prevention strategies has been demonstrated among multiple chronic diseases [24]. WeChat-based DTx overcomes conventional mobile app barriers and drives health care innovation by enhancing patient adherence and care quality across diverse medical contexts, including chronic disease management and preventive strategies.

Previous studies have highlighted the limitations in the current landscape of DTx for fluid management and urinary stone prevention. Stevens et al [25] noted that many apps lack the support of medical professionals, leading to the dissemination of potentially false information and inaccuracies that could negatively impact patient health. This underscores the importance of involving medical professionals in the development and content review of these apps to ensure their reliability and clinical validity. The advent of digital technologies, such as wearable devices and intelligent water bottles, has introduced new methods for monitoring water intake and preventing urinary stones. However, the effectiveness of these devices is highly dependent on the practicality of their use. For instance, patients may not always have access to smart bottles in all settings, which can lead to incomplete fluid intake records. Karagöz and Sarıca [26] further pointed out that the high cost and inconvenience associated with these devices may deter long-term patient compliance, highlighting the need for more cost-effective and user-friendly solutions for fluid intake notification. A review of the literature reveals that most published studies are small-scale, single-arm pilot interventions lacking robust controlled trials designed to evaluate long-term effectiveness and adherence. Moreover, Philip-McKenzie et al [27] analyzed 51 apps from the Apple and Google Play Store and found that none of the apps measured urine volume and color, and only 1 provided medical advice or educational content regarding the consequences of poor hydration. This reveals a substantial gap in the provision of comprehensive medical advice and education within these apps to improve patients’ recognition of disease severity. Furthermore, Winoker et al [28] highlighted the scarcity of evidence supporting the benefits of mobile health (mHealth) interventions in urological care and emphasized the need for more rigorous research to validate the effectiveness of these technologies. Karagöz and Sarıca [26] also recommended that future research should focus on the practicality, security, efficacy, and overall benefits of these apps in preventing stone recurrence.

This study aims to investigate the efficacy of the WeChat-Based Applet Fluid Intake Reminder (WAFIR) in improving fluid intake among postoperative patients with UC compared to standard care, with the ultimate goal of reducing stone recurrence and enhancing patient well-being. The specific objectives are to quantify changes in daily fluid intake and urine output, evaluate its effect on the QoL, and determine its influence on readmission rates, recurrence rates, and eHealth literacy over the follow-up period. We hypothesize that patients receiving WAFIR exhibit statistically significant differences in 24-hour urine output (mL), fluid intake (mL/24 hours), QoL (Wisconsin Stone Quality of Life Questionnaire [WISQOL] score), eHealth literacy (Electronic Health Literacy Scale [eHEALS] score), or urinary stone recurrence rates (3-month follow-up) compared to those receiving usual care.

## Methods

### Research Design

**Figure 1.**
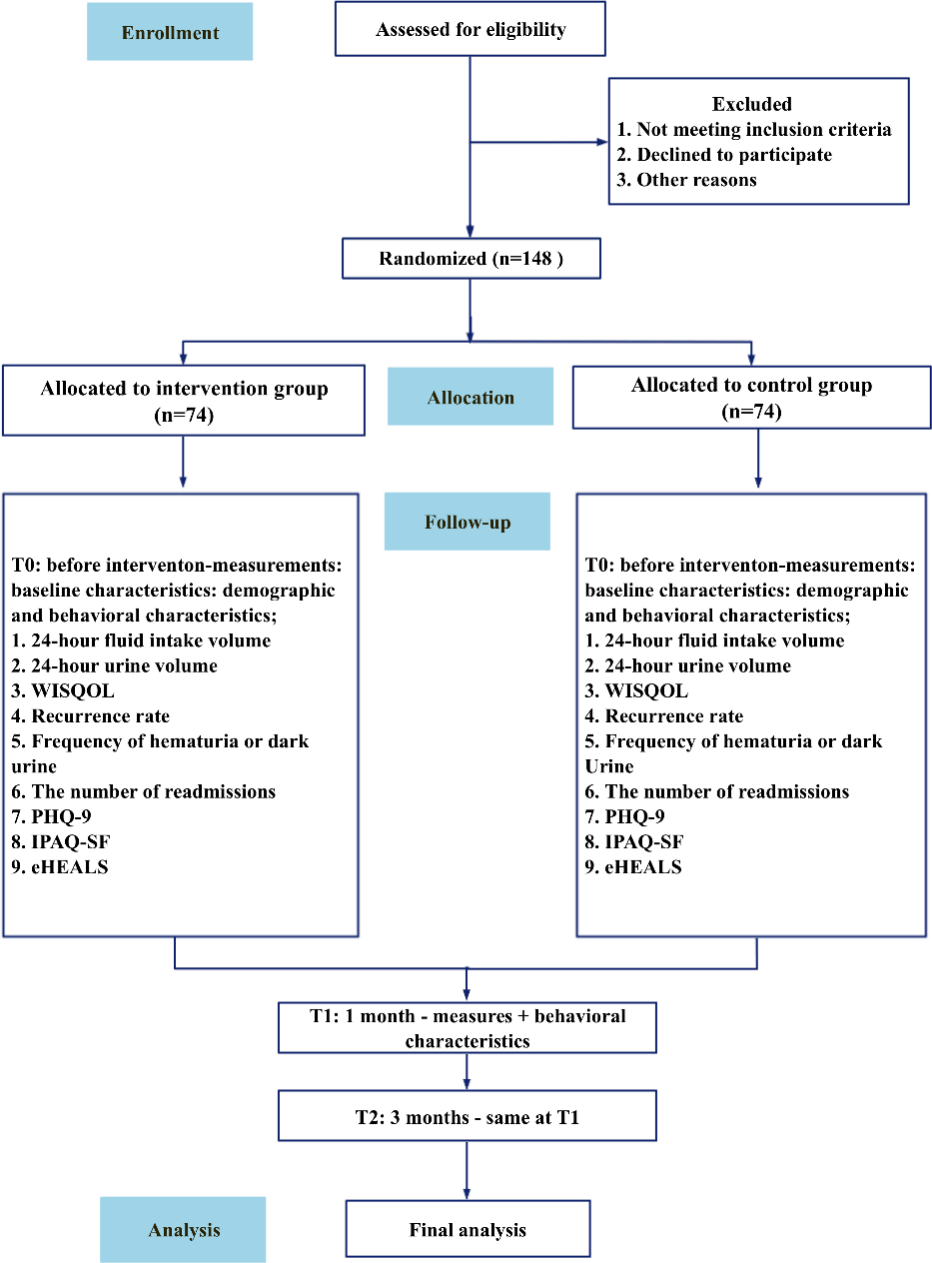
RCT CONSORT flowchart. CONSORT: Consolidated Standards of Reporting Trials; eHEALS: Electronic Health Literacy Scale; IPAQ-SF: International Physical Activity Questionnaire—Short Form; PHQ-9: Patient Health Questionnaire-9; RCT: randomized controlled trial; WISQOL: Wisconsin Stone Quality of Life Questionnaire.

**Figure 2.**
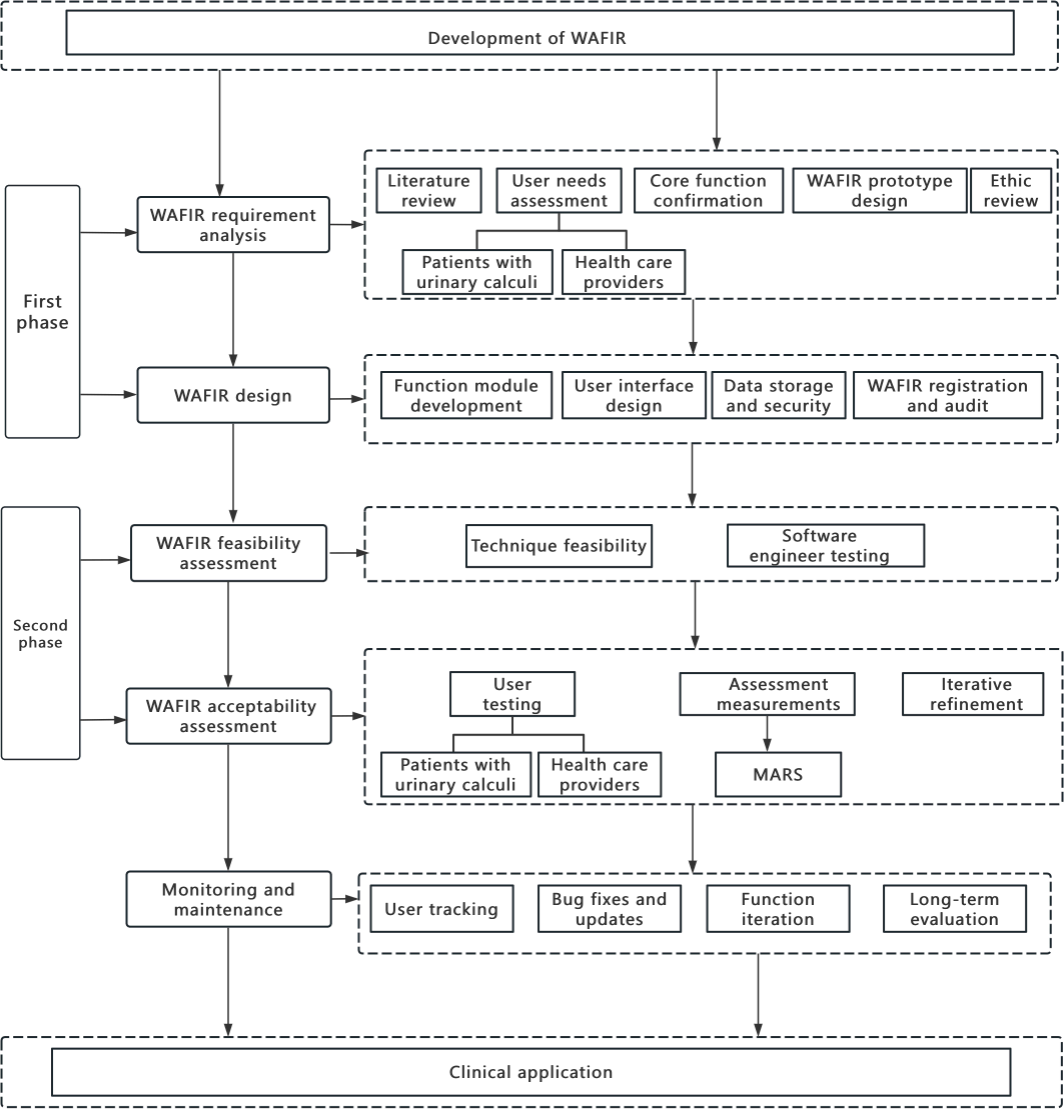
Study design table. MARS: Mobile App Rating Scale; WAFIR: WeChat-Based Applet Fluid Intake Reminder.

We will conduct a 3-month, 2-arm randomized controlled trial (RCT) involving participants assigned to either a WAFIR intervention group or standard care group. Participants will be enrolled in batches, using block randomization with blocks of 10, and allocated in a 1:1 ratio, with a total sample size of 148 participants determined. An independent researcher (QC) will generate the randomization list using the sealed envelope method. A detailed research technology roadmap is displayed in Figure 1. Key aspects of the study design can be found in Figure 2.

### Characteristics of Participants

The study is open to individuals aged 18 years or older, diagnosed with UC and admitted to Shekou Free Trade Zone Hospital (a tertiary general hospital) for urinary stone surgery, to participate. Eligible patients will be enrolled prior to discharge, either by a nephrologist or a urologist (Textbox 1).

Textbox 1. **Inclusion, exclusion, and withdrawal criteria for participants’ recruitment.**

**Inclusion criteria**

- With a history of urinary calculi and having received urolithiasis surgery before.
- 24-hour fluid intake <2500 mL.
- Age ≥18 years.
- Able to use WeChat daily.
- Able to read and write informed consent.

**Exclusion criteria**

- Participants with obstructive uropathy, chronic urosepsis, renal failure, and renal tubular acidosis.
- Participants with congestive heart failure, psychiatric conditions, pregnancy, and primary hyperparathyroidism.
- Participants experienced complicated urolithiasis surgery (surgical procedures for urolithiasis that involve complex interventions, often requiring multiple operations during the follow-up period due to factors such as large stone burden, anatomical abnormalities, or postoperative complications, eg, percutaneous nephrolithotomy with residual stones or ureteroscopy requiring reintervention).
- History of recurrent urinary tract infection (>3 per year).
- Medication use increases stone risk.
- Participants with physical disabilities.

**Withdrawal criteria**

- Participants will be deemed to have withdrawn from the study upon the voluntary withdrawal of their consent or if they fail to return a postintervention assessment within 30 days after the end of the intervention phase. All instances of withdrawal and the discontinuation of any participant’s involvement in the trial are meticulously documented.

### Intervention

#### Standard Care

Participants will receive standard dietary and regular recommendations, including an education profile and counseling to achieve a water intake and urine output ≥2500 mL, along with verbal health education counseling regarding urological calculi prevention during hospitalization and guidance on adequate hydration at discharge. A fixed-capacity water bottle will be provided. Phone calls will be given at each follow-up phase to reinforce the educational content and collect primary and secondary data through questionnaires.

#### WAFIR Intervention

Participants will receive the standard dietary counseling and an educational handout. The WAFIR will be introduced to the intervention group to enhance adherence to fluid intake and facilitate self-monitoring for the prevention of UC. The WAFIR will include features such as fluid intake reminders, fluid and urine value recording, urine color tracking, and health education, as well as an interactive group for communication among postoperative patients with urinary stones. The WAFIR will deliver personalized daily notifications to remind participants of increasing fluid intake exceeding 2500 mL. Additional educational content highlighting the benefits of adherence will be provided to the experimental group through the applet as well. Phone calls will be given at each follow-up phase to collect primary and secondary data via the questionnaires.

To address the limitations observed in existing mobile apps, we are developing an innovative WAFIR aimed at improving participants’ adherence to fluid intake and reducing the recurrence of UC. This intervention will incorporate multiple functionalities to enhance participants’ awareness and control over their fluid consumption. The WAFIR will consist of 4 main pages:

- Homepage: The primary interface of the WAFIR provides a user-centric platform for monitoring and managing daily hydration objectives. It enables patients to set personalized fluid consumption targets, log daily fluid intake, record urine output volumes, and visually assess urine color to determine hydration status.
- Statistical Analysis page: This offers a comprehensive overview of patients’ hydration and urination patterns over multiple time frames. It presents historical data and trends in fluid intake and urine output, displayed on a daily, monthly, and annual basis. Through graphical visualizations, this module empowers patients and health care providers to monitor adherence trends, identify patterns, and adjust intervention strategies to optimize long-term compliance with fluid intake recommendations.
- Health Education Knowledge page: The evidence-based materials tailored to kidney stone prevention are provided, which include detailed information on various types of UC and targeted prevention strategies. This is delivered through accessible formats like articles.
- Function page: The applet will send notification messages at regular intervals, customized based on the patient’s daily habits. The notification module delivers customized reminders designed to promote consistent fluid intake. By sending timely and context-relevant messages, the system encourages adherence to prescribed hydration regimens, addressing barriers such as forgetfulness or situational constraints.

**Figure 3.**
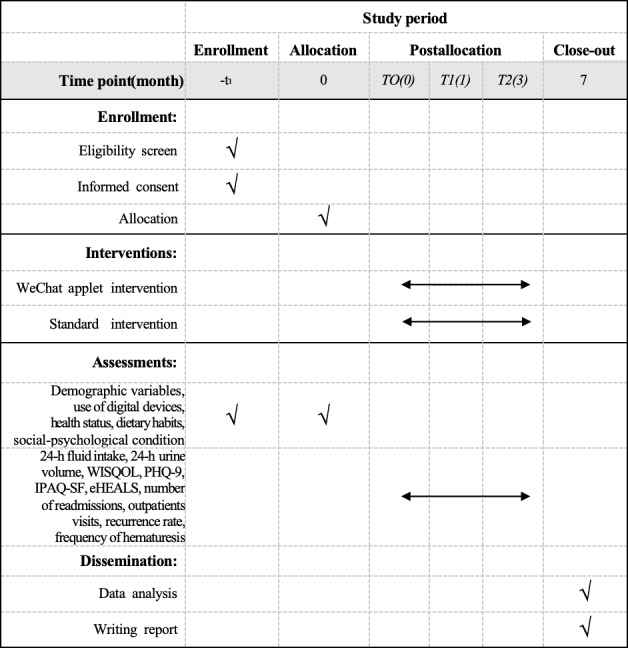
Development of WAFIR flowchart. eHEALS: Electronic Health Literacy Scale; IPAQ-SF: International Physical Activity Questionnaire—Short Form; PHQ-9: Patient Health Questionnaire-9; RCT: randomized controlled trial; WISQOL: Wisconsin Stone Quality of Life Questionnaire.

The development of the WAFIR will begin with its registration on the WeChat Official Accounts platform, initiating a structured 6-phase development process as depicted in Figure 3. The first phase, WAFIR requirement analysis, will involve a multidisciplinary team—including urological nurses, doctors, nursing education specialists, nutritionists, and postoperative patients with UC—to conduct needs assessments, define functional specifications (eg, fluid intake reminders and urine output tracking), and develop evidence-based content through stakeholders’ feedback. The second phase will be the WAFIR design phase, where a user-friendly interface and function module will be created in collaboration with software developers and health care providers. The third phase of the WAFIR feasibility assessment will evaluate the prototype’s technical performance and clinical utility through testing by the development team and clinical professionals. In the fourth phase, the WAFIR acceptability assessment will engage a cohort of 19 participants—comprising 3 urological nurses, 3 urological doctors, 2 nursing education nurses, 1 nutritionist, and 10 postoperative patients—to assess usability and collect feedback using validated tools such as the Mobile App Rating Scale [29] and the mHealth Evidence Reporting and Assessment checklist [30], ensuring that the WAFIR meets patient needs prior to the RCT. The fifth phase, monitoring and maintenance, will focus on ongoing improvements, incorporating user data and feedback to refine content and functionality. Finally, the clinical application phase will implement the fully validated WAFIR in real-world clinical settings for the RCT, aiming to enhance health management and prevention outcomes.

#### Intervention Nurses

The principal investigator (XZ), a clinical nurse on the unit, will train the nursing team on the use of the WAFIR app. Nurses will be involved in securing informed consent from patients by interpreting the purpose, procedures, and anticipated benefits of the study and instructing patients how to use the WAFIR to log their fluid intake. Additionally, they will collect baseline and follow-up data through questionnaires at hospitalization and at 1-month and 3-month intervals. Physicians will evaluate and screen admitted patients to determine whether they meet the inclusion criteria for the research.

#### Intervention Mechanism

**Figure 4.**
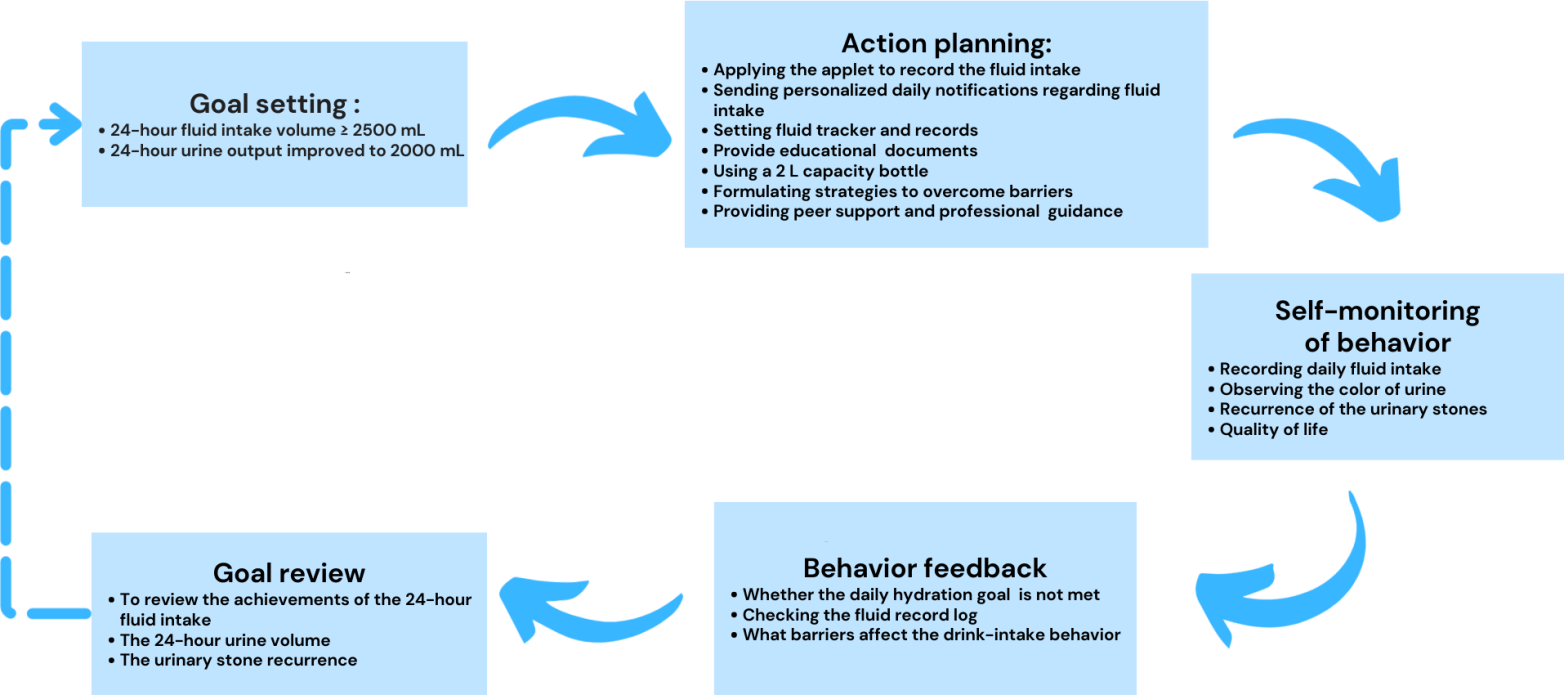
The mechanism of WAFIR. WAFIR: WeChat-Based Applet Fluid Intake Reminder.

The mechanism of the WAFIR provides a robust and constructive framework by providing a feedback loop, which is depicted in Figure 4, to enhance the adherence awareness of fluid intake for patients with UC, which is applied following these processes: goal setting to define the objective and specify the desired outcome: clearly define the water intake goals for patients, such as the amount of drink to be consumed at designated times; begin by establishing long-term and short-term goals. The long-term goal could be to prevent further formation of UC, while a short-term goal could be to ensure a specific amount of water intake per day. Action planning outlines a detailed process on how to achieve the set goals. This could involve developing a WeChat applet monitoring system to track each patient’s water intake, deliver hydration reminders, formulate strategies to overcome barriers to hydration, provide educational documents, facilitate peer support from the community, and offer access to professional guidance. Essential training is also provided to ensure that patients are adept at using the WAFIR to manage and monitor their water intake. Self-monitoring of behavior: encourage patients to monitor their behavior, for instance, recording their daily water intake, the color of their urine, and the physical activity level they perform daily.

Concurrently, educating patients on the importance of proper hydration and its significant impact on health increases their intrinsic motivation. Nurses assist patients with behavioral skills development by setting reminders, recording water intake, and developing strategies to overcome barriers to hydration. Behavior feedback: the WAFIR incorporates feedback mechanisms to provide real-time feedback to patients. This is using notifications, alerts, or visual indicators to signal when patients’ hydration goals are not met. Data collected through the WAFIR are analyzed to recognize patterns of compliance and noncompliance, which help regulate behavior and reduce disparities between current and desired behaviors. This fosters self-monitoring and self-regulation through the WAFIR to enhance patients’ sense of self-efficacy for their drink intake. Goal review: regularly review the goals in light of the monitored behavior and feedback, which may involve adjusting the frequency of reminders, fluid intake goals, physical activity levels, dietary habits, or health literacy to align with patient behavior and feedback. This approach is supported by Partridge et al [31], who demonstrated that mHealth interventions using techniques such as goal setting, self-monitoring of behavior, and feedback are more effective in promoting behavior modification than apps lacking these features. Similarly, a systematic review by Middelweerd et al [32] evaluated 41 apps in iTunes and 23 apps in Google Play, identifying goal setting, self-monitoring, and feedback on performance as common approaches to promote intention to behavioral alteration.

### Theory-Based Technique

**Figure 5.**
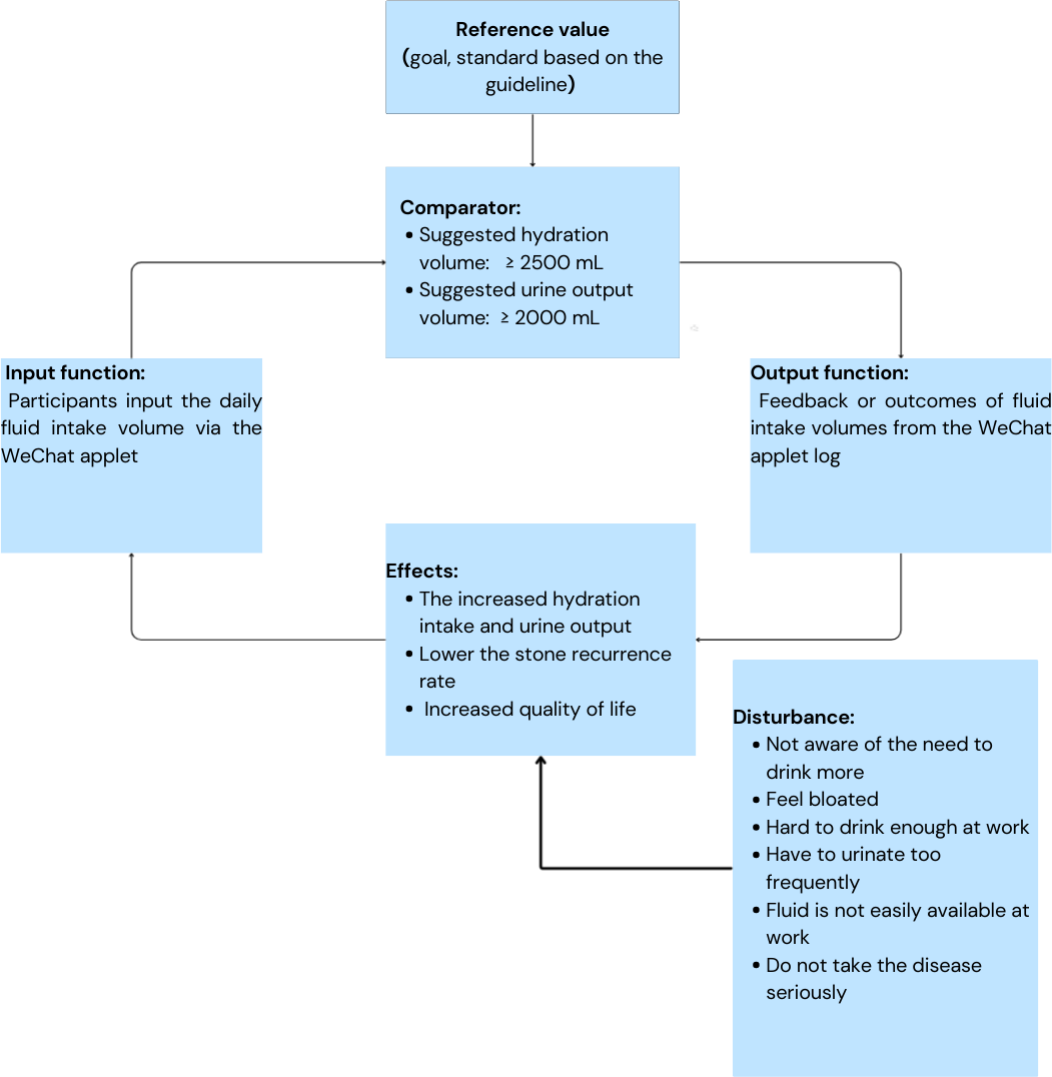
Control theory diagram.

Control theory examines how individuals monitor and modulate their behaviors to attain specific objectives. It underscores the importance of self-regulation, including behavior self-assessment, the establishment of targets, and the use of feedback mechanisms [33]. Within the domain of health behaviors, control theory delineates the strategic adjustment of behaviors to fulfill health-related goals. Meanwhile, control theory, which comprises an input function, a comparator with reference values, an output function, an effect on the environment or others, and disturbance, is majorly applied to bridge the intention-behavior gap in artificial intelligence (AI) for physical action change. The detailed diagram described in Figure 5 closely aligns with the WAFIR mechanism. The goal-setting and action-planning stages correspond to the input function and comparator phases; the self-monitoring of behavior and behavior feedback phases can be reflected in the output function; and the goal review stages can be found in the effects phase of the control theory, with disturbances identified accordingly.

### Ethical Considerations

This study received ethics approval from the University of Hong Kong/Hospital Authority Hong Kong West Cluster Ethics Review Board (UW24-754) and the Qianhai Shekou Free Trade Zone Hospital Ethics Committee (2024-055(Shen)-01) in December 2024. Written informed consent will be obtained from all participants, ensuring their right to withdraw, per the Declaration of Helsinki. Participant data will be deidentified, stored securely, and accessed only by authorized personnel to ensure privacy and confidentiality. The trial does not involve physical harm. No compensation will be provided, with voluntary participation incentivized by potential health benefits. No identifiable images or information will be included in the manuscript or supplementary materials. All procedures adhere to institutional and international ethical standards.

### Trial Registration

Our trial was retrospectively registered on ClinicalTrials.gov (NCT06990672) on May 24, 2025, after the initiation of recruitment. This delay was unintentional and resulted from unforeseen administrative challenges, including delays in securing institutional registration and coordinating with the tertiary hospital’s research office in Mainland China, which were exacerbated by the rapid progression of the study timeline following the development of the WAFIR applet in February 2025. Recruitment commenced on March 1, 2025, and was completed by June 27, 2025, prior to registration, due to these logistical constraints. We affirm that this retrospective registration does not reflect an intent to bias reporting, as the study protocol, including design, outcomes, and intervention details, was finalized and documented internally before recruitment began, adhering to the original research plan.

### Outcome Measures

#### Primary Outcomes

##### 24-Hour Urine Volume

To initiate the 24-hour urine collection, upon waking in the morning, void into a graduated container and document the precise start time (eg, 8 AM). On the subsequent morning, near the 24-hour end point (eg, 8 AM), we record the urine volume once more. Throughout this period, we use platforms such as the WeChat applet or alternative recording methods to track and document the cumulative urine output.

##### 24-Hour Fluid Intake Volume

This measure is to calculate the participants’ fluid intake for a full 24-hour period. This comprehensive record should span a full 24-hour period, ideally starting and ending at the same time of day to standardize the assessment. Participants will be instructed to record the fluid consumed using either the WAFIR or another recording method, including water, tea, coffee, Chinese herbal tea, and other eligible liquids. The data on daily fluid consumption volume can be summarized automatically once patients log the data in the WAFIR in the intervention group. However, the participants must manually report the fluid intake volume in the control group. This is the direct parameter that reflects the adherence to fluid intake in the WAFIR.

### Secondary Outcomes

#### Wisconsin Stone Quality of Life Questionnaire

Penniston et al [34] developed the WISQOL as a disease-specific measure to assess the QoL of patients with UC. It encompasses 7 dimensions: vitality and fatigue, sleep, work and family, nutrition (diet) and pharmacotherapy, physical conditions, concerns over intimacy and traveling, and emotional status in general [34]. The scale consists of 28 items, each rated on a 5-point scale from 1=very true to 5=not at all true, yielding a total score range between 28 and 140. A higher score indicates a better QoL. This scale is widely used in multicenter studies in various countries, such as the United States and Canada, for its well-confirmed reliability and validity. Cronbach α coefficient was 0.948, and the reliability was 0.907 in the Chinese version.

#### Patient Health Questionnaire-9

This assesses participants’ mental health status after receiving different interventions, which can help monitor the health status and psychological well-being of patients [35]. Nine items with a cumulative score on the Patient Health Questionnaire-9 (PHQ-9) range from 0 to 27. Cronbach α coefficient was 0.839, and the reliability was 0.846 in the Chinese version.

#### International Physical Activity Questionnaire—Short Form

This assesses the participants’ physical activity levels and the frequency and duration of the workout [36], which can affect the fluid requirements and the risk of UC formation. The questionnaire consists of 7 questions to assess physical activity level over the past week. Activity is classified as low if the total score is <600 metabolic equivalent of task-minutes per week and classified as high if it is >3000 metabolic equivalent of task-minutes per week. The intraclass correlation coefficients for all physical activity categories within the International Physical Activity Questionnaire were above 0.7 in the Chinese version.

#### Electronic Health Literacy Scale

This scale is designed by Norman and Skinner [37] to measure an individual’s self-perceived skills in using information technology for seeking, searching, understanding, and appraising health information from electronic sources. Including 8 items with a 5-point Likert scale, the total score ranges from 8 to 40. The Cronbach α coefficient of the eHEALS scale is 0.963. The retest reliability coefficient *r* was 0.984 (*P*<.001) in the Chinese version.

#### The Number of Readmissions, Outpatient Visits, and Emergency Department Visits

This assesses the frequency of hospital readmissions related to UC, including the timing of such readmissions due to stone recurrence or related symptoms (eg, hematuria, pain, and infection) and the number of outpatient visits in terms of UC management. The frequency of emergency department visits is due to UC. Any medications might affect fluid intake or urinary output or improve the symptoms that triggered postoperatively.

#### Recurrence Rate

The rate at which new UC forms during the 3-month follow-up.

#### Frequency of Hematuria or Dark Urine

The frequency of the presence of blood in urine or dark urine, which can be a symptom of a new urinary stone or a signal of less fluid intake.

### Sample Size

An adequate sample size and statistical power ensure that the findings can be reliably generalized to a broader population. The required sample size for hypothesis testing is determined based on the expected effect size [38]. In this study, the sample size will be determined by a statistical tool, G*Power software (Heinrich Heine University Düsseldorf) [39], with an α of .05, a power of 0.80, and an allocation ratio of 1:1. The sample size for this study was calculated based on a comparable RCT that used an eHealth device (an intelligent water bottle) to promote increased fluid intake [18], of which the calculated Cohen *d* was 0.61, and the effect size is considered significant since Cohen *d* is over 0.5. Based on the calculations, a sample size of 92 participants is estimated. Accounting for an attrition rate of 38%, as observed in this research, a total of 148 participants are anticipated to be recruited.

### Randomization, Blinding, and Concealed Allocation

An RCT will be conducted to recruit participants in a single site, the Shekou Free Trade Zone Hospital. Randomization may lead to baseline characteristic imbalances between intervention groups [40]. In small-scale trials, stratification might be beneficial, as it can prevent significant imbalances in factors predicting outcomes [41]. Stratified randomization will be applied to the eligible participants to ensure an equal chance to be allocated to the research field [42]. Balanced randomization aims to create unbiased groups for comparison and also seeks to maintain groups of roughly equivalent size throughout the trial [43]. In this research, convenience sampling will be applied, with block randomization applied using a block size of 10. For every group of 10 participants enrolled in sequence, 5 will be allocated to the intervention group and the remaining 5 to the control group, which will be done by the statistician and urologists who are not engaged in conducting the research interventions.

### Data Collection

The study will be conducted on postoperative patients with UC. It will span 3 periods: T0 (before the intervention), T1 (at the end of 1 month), and T2 (at the end of 3 months). The data collected at each time point will allow for evaluating the intervention’s effectiveness and identifying areas for improvement. Reminders will be sent prior to each follow-up visit using the participant’s preferred method of communication, with a thank-you message after the visit. For any participant who discontinues the intervention prematurely, we will record the reason for their discontinuation and collect any serious adverse events that occurred up to the point of discontinuation.

### Methods of Analysis

The study outcomes will be reported to adhere to the guidelines outlined in the CONSORT (Consolidated Standards of Reporting Trials) 2010 statements [44]. Statistical tests such as a 2-tailed *t* test and chi-square test will be used for continuous and categorical variables within 2 groups. The principal analysis will compare the fluid intake volume between the intervention and control groups using the 2-tailed *t* test. Secondary outcomes such as WISQOL, PHQ-9, and eHEALS will use repeated measures of ANOVA since they are measured over time and between 2 groups. If necessary, subgroup analyses will be conducted to explore whether the effect of the intervention varies across different demographic or clinical subgroups. Intention-to-treat analysis will be used to assess the impact of the treatment and its generalizability as well as the missing data. A planned interim analysis will be performed to evaluate both efficacy (whether the intervention shows sufficient benefit to potentially stop the trial early) and futility (whether the intervention is unlikely to demonstrate benefit if the trial continues) after 50% (74/148) of the target sample size have completed the primary end-point assessment. A significance level of *P*<.05 will be used to determine statistical significance, and all data analysis procedures will be conducted using the SPSS (version 26.0; IBM Corp) software package.

### Access to Data

The researcher is granted access to the complete trial dataset, with a contractual agreement established with the statistician to manage the data confidentially in accordance with the research protocol.

## Results

This research has achieved notable advancements in assessing the efficacy of a nurse-led digital health intervention, WAFIR. The development of WAFIR, conducted in collaboration with a software developer, was finalized in February 2025, establishing a technological platform for the study. Recruitment, a critical initial stage, was fully completed, with all 148 participants enrolled from a tertiary hospital in Mainland China between March 1, 2025, and June 27, 2025, exclusively during the predischarge hospitalization period. Baseline data collection (T0) was completed by June 30, 2025, providing a solid baseline for longitudinal analysis. Follow-up assessments at the 1-month mark (T1) were finalized on July 31, 2025, while the 3-month follow-up (T2) is set to conclude in October 2025, aligning with the present date. Subsequent data analysis is scheduled for completion by December 2025, with dissemination of results planned through academic conferences and peer-reviewed publications throughout 2026.

## Discussion

This study represents, to our knowledge, one of the first RCTs to assess a nurse-led, WeChat-based DTx specifically tailored for UC management, addressing a critical gap in mHealth interventions that often lack medical oversight or clinical validation. The primary outcomes of this study are to evaluate the effectiveness of the WAFIR in improving fluid intake and urine output among postoperative patients with UC. We hypothesize that the WAFIR intervention, combining fluid intake reminders, urine color monitoring, fluid and urine tracking, and evidence-based educational content, will significantly enhance adherence to hydration recommendations compared to standard care, potentially lowering recurrence rates and improving patient-reported outcomes such as QoL (via WISQOL), eHealth literacy (via eHEALS), and mental health (via PHQ-9).

This study used face-to-face and telephone interviews to collect data. However, interviews are subject to limitations, including the absence of visual cues (particularly in telephone interviews), reduced ability to observe contextual details, and potential difficulties in eliciting sensitive information, which may affect data quality [45]. The primary outcome, 24-hour urine volume, is required to be collected. However, previous studies indicate challenges in obtaining precise 24-hour urine samples due to poor patient compliance, which may result in incomplete or abandoned collections stemming from the complex or inconvenient nature of the process, as well as deviations from the 24-hour collection period. To enhance the measurement accuracy of 24-hour urine collection, it is crucial to improve patient education on the importance of urine collection; simplify the collection process with clear guidelines, flowcharts, and user-friendly containers; and provide necessary tools like graduated urinals or collection bags. Regular monitoring and feedback sessions are essential to address any issues promptly. Additionally, incentivizing participation with rewards can boost patient motivation, ensuring higher compliance and accurate urine volume measurements within the specified time frame.

A key strength of this study is its rigorous RCT design, with clear inclusion criteria, randomization, and intention-to-treat analysis, ensuring robust evidence generation. However, there are several limitations to this protocol that should be acknowledged. First, patients were recruited from a single site. This limitation may affect the generalizability of the findings, as the sample may not be representative of the broader population. Second, the current WAFIR is equipped with a fluid record function, evidence-based educational content delivery, automated reminders and notifications, and data collection and reporting. AI technologies, such as AI-driven predictive models, are not equipped within the WAFIR, as the integration could enhance its effectiveness by providing tailored health education, motivational messages, and personalized recommendations based on individual patient data and behavior patterns.

The dissemination plan includes presenting research findings at international conferences and forums and publishing in peer-reviewed journals in 2026. If proven effective, WAFIR could establish a pathway for developing other nurse-led digital interventions, offering a low-cost and scalable approach to enhance patient outcomes in chronic disease management. Furthermore, the findings will guide app developers in creating clinically validated mHealth tools and assist clinicians in integrating such interventions into standard urolithiasis care, thereby potentially transforming postoperative management practices.

## Abbreviations

AI: artificial intelligence
CONSORT: Consolidated Standards of Reporting Trials
DTx: digital therapeutic intervention
eHEALS: Electronic Health Literacy Scale
mHealth: mobile health
PHQ-9: Patient Health Questionnaire-9
QoL: quality of life
RCT: randomized controlled trial
UC: urinary calculi
WAFIR: WeChat-Based Applet Fluid Intake Reminder
WISQOL: Wisconsin Stone Quality of Life Questionnaire
